# E-cigarette exposure disrupts antitumor immunity and promotes metastasis

**DOI:** 10.3389/fimmu.2024.1444020

**Published:** 2024-08-16

**Authors:** Marcel Arias-Badia, Chien-Chun Steven Pai, PeiXi Chen, Anthony Chang, Yee May Lwin, Aahir Srinath, Jeffrey E. Gotts, Stanton A. Glantz, Lawrence Fong

**Affiliations:** ^1^ Division of Hematology/Oncology, Department of Medicine, University of California, San Francisco, San Francisco, CA, United States; ^2^ Kaiser Permanente San Francisco Medical Center, San Francisco, CA, United States; ^3^ Center for Tobacco Control Research and Education, University of California, San Francisco, San Francisco, CA, United States; ^4^ Helen Diller Family Comprehensive Cancer Center, University of California, San Francisco, San Francisco, CA, United States; ^5^ Division of Cardiology, Department of Medicine, University of California, San Francisco, San Francisco, CA, United States; ^6^ Fred Hutchinson Cancer Center, Seattle, WA, United States

**Keywords:** electronic cigarettes, metastasis, whole body exposure, immunosuppression, immune checkpoint blockade

## Abstract

Electronic cigarettes (e-cigarettes) are thought to pose low risk of cancer because the components of e-cigarette liquid are not carcinogens. We analyzed the effects of the two major components, PG/VG and nicotine, on tumor development in preclinical models. We found that PG/VG promoted tumor cell migration in migration assays and contributed to more aggressive, metastatic, and immunosuppressive tumors *in vivo*, aggravated by the presence of nicotine. Whole body exposure of mice to PG/VG and nicotine rendered animals more susceptible to developing tumors with high frequencies of infiltrating proinflammatory macrophages expressing IL-6 and TNFα. Moreover, tumor-infiltrating and circulating T cells in e-cigarette exposed mice showed increased levels of immune checkpoints including CTLA4 and PD-1. Treatment with anti-CTLA4 antibody was able to abrogate metastasis with no detrimental effects on its ability to induce tumor regression in exposed mice. These findings suggest that the major components used in e-cigarette fluid can impact tumor development through induced immunosuppression.

## Introduction

E-cigarettes deliver nicotine to users by aerosolizing a solution of propylene glycol, glycerin, nicotine and flavoring agents. While e-cigarette use has been linked to cardiovascular, metabolic, pulmonary, oral and other diseases in people, the effects of e-cigarettes on cancer in people is limited (1). Because they do not burn tobacco, e-cigarettes generate much lower levels of combustion-related carcinogens than cigarettes do (2). It is also widely noted that nicotine is not a carcinogen. Propylene glycol and vegetable glycerin (PG/VG) (3) are seen as benign because they are widely used as humectants in food and are generally recognized as safe for ingestion. As a result, e-cigarettes are widely assumed to impose minimal cancer risks (4). Indeed, e-cigarettes have been promoted as a harm reduction alternative to cigarettes among smokers with cancer (5–7).

While not a carcinogen, nicotine has been associated with tumorigenesis in a number of malignancies, mainly through engagement of nicotinic acetylcholine receptors (8, 9). Nicotine can also induce malignant cell cycle transformation through a number of pathways (10–12), including alteration of p53 function (13). Nicotine also has immunomodulatory effects, including suppression of T cell proliferation *in vitro (* 14), engagement of inhibitory pathways in the form of immune checkpoints like PD-1, and blunting of downstream IL-2 signaling in CD8+ T cells (15). Additionally, nicotine has been shown to induce immunosuppressive tumor microenvironments by means of proinflammatory cytokine upregulation (16) or recruitment of protumoral myeloid populations such as M2 macrophages or N2 neutrophils, thereby promoting metastasis and hampering antitumor immunity both in cancer- and immune-intrinsic manners (17–19).

PG/VG, which is not a carcinogen, induces proinflammatory cytokines and extracellular matrix components that contribute to tumor angiogenesis, an effect increased by nicotine (20). *In vivo* data shows that PG/VG caused T cell immunosuppression and proinflammatory cytokine release, with and additive effect from nicotine (21). Based on these effects, one would expect that exposure to a mixture of PG/VG and nicotine would promote the growth and metastasis of established tumors. Indeed, Pham et al. found that e-cigarette exposure promoted breast cancer and lung metastasis in mice (19). The present study expands this work to assess the *in vitro* and *in vivo* effects of exposure of cancer cells to PG/VG and nicotine. We found that exposure of tumor cells to PG/VG and nicotine increased metastases. We also found an immunosuppressive effect upon e-cigarette exposure in the myeloid and lymphoid tumor-infiltrating compartments, including induction of exhaustion markers on T cells. Finally, we show that these exhaustion markers are functional as the tumors could respond to immune checkpoint blockade (ICB). In sum, although not carcinogens, PG/VG and nicotine promote the progression of established cancers.

## Materials and methods

### E-cigarette liquids

E-cigarette liquids were prepared the same for *in vitro* exposure of cell cultures and *in vivo* whole-body exposure of mice. The PG/VG solution was prepared by mixing PG (99.5% USP grade, CAS 7–55-6, density 1.26 g/ml) and VG (99.7% USP grade, CAS 56–85-1, density 1.04 g/ml) (both from MyFreedomSmokes, https://myfreedomsmokes.shop/) at a 1:1 volume ratio. Nicotine (N3876, Sigma) was added to the PG/VG mix to the desired experimental concentration (0, 6 and 36 mg/ml). All solutions were sterile-filtered through 0.45 μm filters before exposure to cells or mice.

Preconditioning of tumor cells+ in culture prior to subcutaneous challenge was performed for 14 days at 37 C in 6-well plates, with control groups being incubated only in glucose, pyruvate-supplemented Dulbecco’s Modified Eagle Medium (DMEM) containing 10% fetal bovine serum for the same amount of time. Conditioned media were replaced every 2 days.

### *In vitro* scratch assays

To assess the impact of PG/VG and nicotine on tumor cell migration, scratch assays were performed by adapting a protocol described elsewhere (22). Briefly, freshly passaged colorectal carcinoma MC38 cells were rested overnight at 37C. Then, 7 x 10^4^ cells were seeded into each side of 2-well coculture inserts containing a ‘scratch’ or cell-free gap (80209, Ibidi) and incubated at 37C overnight. Then, after checking that a monolayer was formed inside the inserts, the inserts were removed with sterilized forceps, cells washed once with Phosphate Buffered Saline (PBS), then culture media added containing PG/VG and nicotine, and plates incubated at 37C. PG/VG alone was dissolved in PBS at concentrations ranging from 2.5 to 20 μM. In wells exposed to PG/VG + nicotine, PG/VG was kept at 2.5 μM for all nicotine concentrations. Cell migration was then monitored by time lapse microscopy (Incucyte). Migration rates were quantified through Incucyte built-in confluency calculator as percentage of scratch area -defined by a diameter between the fronts of each monolayer- covered by cells. After 7 days in culture, viable cells were quantified in a Vi-CELL counter (Beckman Coulter).

### Animal studies, cell lines and *in vivo* imaging

8- to 10-week-old male wildtype C57BL/6J mice (000664, Jackson) were used in mouse experiments. All cell lines used in this study were certified yearly by the STR Profiling method available through ATCC at https://www.atcc.org/services/cell-authentication/mouse-cell-str-testing. Latest certification date was January 2023. We conducted both systemic and subcutaneous *in vivo* tumor studies. In systemic studies, tumor cells are injected via tail vein into bloodstream. They home to lungs to generate ‘systemic tumors’. In subcutaneous studies, tumor cells are injected intradermally on the flank of mice, where they settle and form a solid tumor mass. For subcutaneous *in vivo* tumor studies, 0.25 million untreated or pretreated murine melanoma B16, 0.5 million colorectal carcinoma MC38, or 1 million prostate adenocarcinoma TRAMP-C2 cells were injected on the right flank of mice and tumors were measured twice a week with a digital caliper (Fisher). Tumor volume was obtained with the formula V = L (length) × W (width) × W × π/6, where L was the higher measure, and W was the lower measure. Endpoint volume was set at 2,000 mm^3^.

For systemic tumor experiments, 5 x 10^5^ luciferase-expressing murine colorectal carcinoma MC38 cells (kindly donated by Jeff Bluestone) were injected intravenously through the tail vein of each animal (n=5) on day 0. In preconditioning experiments, cells were harvested from 6-well plates after the 14-day preconditioning plus 1–2 post-thaw passages. In whole body exposure experiments, cells were harvested after 1–2 post-thaw passages. In both cases, cells were counted with a Vi-CELL cell counter (Beckman Coulter) at a 1:60 dilution and prepared at 5M cells/ml in DPBS, 100 µl of which were used for intravenous injections per animal. Tumor progression was monitored using an IVIS Spectrum *in vivo* imaging device (Xenogen). Briefly, animals were injected i.p. with 0.2 ml of DPBS containing 3 mg D-Luciferin (88294, Thermo) and were imaged after a 3-minute incubation. Images were processed and bioluminescence radiance was quantified using Living Image version 4.7.4.20726.

In immune checkpoint blockade (ICB) studies, animals were implanted with 0.5 million MC38 cells, were randomized into treatment groups and were treated intraperitoneally on days 3, 6 and 9 with 200 μg isotype control (IgG2k/a, BioXCell) or anti-CTLA4 (clone UC-10, BioXCell).

All mice were maintained at the UCSF vivarium and received food and water *ad libitum*.

### Whole body e-cigarette exposure

As described previously (23), to assess the impact of inhaled e-cigarette aerosol in the tumorigenesis of systemically implanted tumors, mice were exposed in a chamber to e-cigarette mixes containing PG/VG and 0, 6 or 36 mg/ml nicotine for 1h daily (5 days/week) for 4 weeks, a scheme that has been already reported to show differences *in vivo (* 24). In particular, 36 mg/ml yielded nicotine concentrations similar to that of a standard cigarette smoking model (23). E-cigarette liquids were aerosolized with an atomizer designed for vaping oils (1.8 Ω cotton coil, Aspire) using a Gram Universal Vaping Machine (Gram Research) at 9.4 watts. It was operated with Gram VM software, version 4.15.25. Coil power was set at 4V. Puff volume was 80 ml, drawn over 4 seconds into a syringe through an electronically controlled three-way valve, then injected to vaping chamber over 2 seconds. In order to fill the vaping chamber with aerosol, 10 puffs were initially injected over approximately one minute, followed by 110 puffs over one hour. The chamber was evacuated at a constant rate of 2.0 liters/minute during the exposure using a calibrated flowmeter (Dwyer, Michigan City, IN, US) to draw in a mixture of fresh aerosol and room air. Upon completion of 110 puffs, the vacuum outflow speed was increased to clear the chamber over a period of 5 minutes. After exposure, mice were removed from the chamber. After 4 weeks, whole-body exposure was ceased and 5 x 10^5^ Luc-MC38 cells were injected intravenously onto mice and tumor growth was monitored by *in vivo* bioluminescence as described above. Air-exposed animals were included as control.

### Tissue processing

Lungs and spleens from surviving whole body exposure animals were surgically removed from three mice with sterilized equipment 22 days after tumor injection. These mice were not considered for survival analysis. Lungs were weighed and perfused with PBS. Metastatic nodules were counted. Then, they were mechanically dissociated with scalpel blades and digested to single cell suspensions by incubation for 1h at 37C in tumor digestion media containing DMEM, 10%FBS, 2 mg/ml Collagenase IV (C5138, Sigma-Aldrich) and DNAse I (D5025, Sigma-Aldrich). Spleens were mechanically dissociated through a 70 μm filter into a 50 ml conical tube with cold PBS. Lung and spleen lysates were filtered through a 100 μm filter into 50 ml conical tubes and filled with cold PBS, followed by centrifugation at 450 g for 5 minutes at 4C. Supernatants were discarded and pellets were resuspended in 5 ml ACK Lysing Buffer (118–156-101, Quality Biological), mixed well and kept on ice for 5 minutes. Lysis was stopped by filling the tubes with cold PBS. Samples were centrifuged again and finally resuspended in 1 ml cold PBS. Viable cells for downstream use were counted in a Vi-CELL cell counter (Beckman Coulter) at a 1:60 dilution.

### Flow cytometry

Single cell suspensions were first incubated with 1:100 Zombie NIR (L34976, Life Technologies) for 10 minutes in the dark at room temperature. After washing, surface staining including 1:50 Fc Block (70–0161-U500, Tonbo Biosciences) was performed for 30 minutes on ice. Cells were fixed using the eBioscience FoxP3 kit (00–5523-00, Life Technologies) and intracellular staining was added to samples for 30 minutes on ice before final wash with FACS Buffer (PBS containing 2% FBS and 1mM EDTA). Samples were run on a LSR Fortessa X-50 (Becton Dickinson). Data were analyzed by FlowJo 10.7 (Tree-Star). A detailed list of mouse antibodies used can be found in **Supplementary Table 1**.

For exhaustion marker co-expression, Boolean gating of CTLA4, TIM3, LAG-3 and PD-1 expression was applied on gated splenic CD8+ T cells at FlowJo.

### Data analysis and statistics

Most results were analyzed using regression with PG/VG concentration or presence (coded 0/1 for no/yes) and nicotine concentration (in mg/mL) or presence (coded 0/1) using Stata 15 *mixed*. Tumor growth curves were also analyzed on ln(tumor volume) using *mixed*, including PG/VG and nicotine interactions with time (to allow for slope changes) and nesting days with mouse to account for repeated measures. A quadratic term for time (days^2^ was included the regressions to allow for curvature over time to improve residual patterns). Estimates were computed using restricted maximum likelihood (REML) and unstructured covariance matrices.

The presence of metastases was analyzed for all cell types using a single logistic regression including variables for PG/VG (0/1), nicotine concentration divided by 36 mg/mL (so 1 corresponded to 36 mg/mL), and two effects-coded dummy variables to allow for differences in cell type.

Metastases rates in anti-CTLA4 experiments were analyzed by Fisher Exact tests.

Kaplan-Meier survival curves were analyzed using Mantel-Cox log rank tests in GraphPad Prism 10.1.2, first comparing all groups, then only comparing the PG/VG and nicotine groups.

### Ethics statement

All experiments involving animals performed in this study were reviewed and approved by the Institutional Animal Care and Use Committee (IACUC) at UCSF under protocol number AN202446.

### Data access statement

All data presented in this manuscript and its related **Supplementary files** are available from the corresponding author upon reasonable request.

## Results

### PG/VG promotes tumor cell motility while nicotine attenuates tumor cell duplication *in vitro*

**Figure 1:**
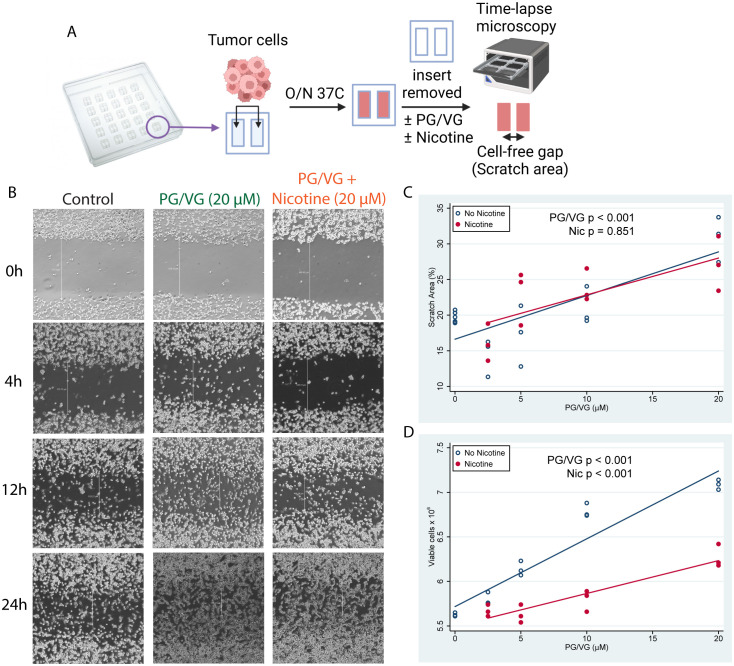
E-cigarette chemicals promote tumor cell migration *in vitro.* **(A)** Experimental design for cell scratch assays. Briefly, tumor cells were seeded into inserts containing a cell-free gap (scratch area) between two sides and were cultured overnight in the presence of concentrations of PG/VG from 0 to 20 µM with or without nicotine. Then, inserts were removed and invasion of the scratch area was monitored by time-lapse microscopy. **(B)** Representative microscope images (40X augmentation) taken at 0, 4, 12 and 24h from 1 of 3 experiments are shown. Vertical white lines show distance from cell fronts in μm, used to calculate percentage of covered scratch areas. **(C)** Percent of scratch area covered at 24h after insert removal fell by 0.58 (95% CI 0.39, 0.77) %/µM PG/VG (p<.001; **Table 1**) but was not affected by the presence of nicotine (p=.851). We also ran the model including the PG/VG x nicotine interaction and found no significant interaction (p=0.638). **(D)** Viable cell counts after 7 days increased by 0.060 (0.048, 0.071) x10^6^ cells/µM PG/VG (p<.001; **Table 1**) and dropped by -0.56 (-0.72, -0.39) x 10^6^ in the presence of nicotine (p<0.001). We also ran the model including the PG/VG x nicotine interaction and found a significant interaction (P<0.001). The PG/VG effect was about the same 0.076 (0.064, 0.088) (p<.001), whereas the nicotine main effect dropped to -0.22 ± .11 (-0.42, -0.02) (p=.029) with the interaction term being -.039 (-0.057, -0.021) (p<.001).

To test whether e-cigarette PG/VG and nicotine affect tumor cell migration *in vitro*, we adapted a protocol developed by Liang and colleagues (22) and exposed adjacent monolayers of colorectal MC38 tumor cells, separated by a cell-free gap or ‘scratch area’, to e-cigarette chemicals, and quantified cell invasion into the scratch area over time (**Figure 1A**, **Supplementary Figure S1** and **Table 1**). Exposure to PG/VG showed a significant dose-dependent increase in the ability of tumor cells to occupy the scratch area after 24h, independent of the presence of nicotine (**Figures 1B, C**). After 7 days of culture, PG/VG without nicotine also showed a significant dose-dependent increase in the number of tumor cells. However, the addition of nicotine led to reduced absolute number of viable tumor cells (**Figure 1D** and **Supplementary Figure S1**). These results indicate that PG/VG can be a promoter of tumor cell proliferation and invasiveness *in vitro*, with nicotine partially blunting proliferation while not affecting tumor cell motility.

**Table 1:** Tumor characteristics.

	Est.	95% CI	Est.			95% CI	p	Est.	95% CI	p
**Figure 1**	Constant (Control) (%)		PG/VG (/µM) [range 0–20 µM]			Nicotine (%) [no/yes]		
Scratch area covered (%) [Panel C]	16.85	(14.79, 18.91)	0.58			(0.39, 0.77)	<0.001	.26	(-2.48, 3.00)	0.851
–Including interaction -0.096 (-0.493, 0.302), p=0.638	16.62	(14.33, 18.92)	0.61			(0.37, 0.86)	<0.001	1.05	(-3.24, 5.33)	0.632
Viable cells x 10^6^ [Panel D]	5.84	(5.70, 5.98)	0.060			(0.048, 0.071)	<0.001	-0.56	(-0.72, -0.39)	<.001
–Including interaction -0.039 (-0.057, -0.021), p<.001	5.71	(5.60, 5.84)	0.076			(0.064, 0.088)	<0.001	-0.22	(-0.42, -0.02)	0.029
**Supplementary Figure S3**			PG/VG [no/yes]			Nicotine (/mg/ml) [0, 6, 36 mg/ml]		
Tumor bioluminescence on day 15 (p/s/cm^2^/sr)	935	(409, 1462)	1232			(567, 1897)	<0.001	13.0	(-6.3, 32.3)	0.187
**Figure 4**			PG/VG [no/yes]			Nicotine [no, yes (36 mg/ml)]		
Tumor bioluminescence on day 14 (p/s/cm^2^/sr) [Panel C]	2985	(2257, 3713)	1617			(588, 2646)	0.002	857	(-172, 1886)	0.103
Metastatic nodules on day 22 (number) [Panel F]	61	(48, 74)	59			(41, 77)	<0.001	86	(68, 103)	<0.001
Lung weight on day 22 (mg) [Panel G]	946	(731, 1162)	-76			(-390, 239)	0.637	400	(86, 714)	0.013
**Figure 5**										
Lung infiltrating macrophage frequency within CD45+ immune cells (%) [Panel A]	38.6	(28.9, 48.2)	6.1			(-7.9, 20.2)	0.392	24.5	(10.4, 38.5)	0.001
CD8+ T cell within CD45+ immune cells (%) [Panel B]	3.93	(3.74, 4.12)	-1.70			(-1.96, -1.43)	<0.001	-0.53	(-0.79, -0.27)	<0.001
IL-6 in lung macrophages [Panel E]	442	(140, 743)	388			(-39.1, 814.1)	0.075	2270	(1844, 2697)	<0.001
TNFα in lung macrophages [Panel F]	0	(-211, 211)	100			(-199, 399)	0.512	4135	(3586, 4434)	<0.001
PD-1 in lung CD8+ cells [Panel I]	20.1	(17.8, 22.3)	62.2			(59.0, 65.4)	<0.001	-1.6,	(-4.7, 1.6)	0.335
TNFα in lung CD8+ cells [Panel J]	964	(862, 1066)	40			(-104, 185)	0.586	2963	(2814, 3112)	<0.001
**Figure 6A**			PG/VG [no/yes]			Nicotine [no, yes (36 mg/ml)]		
CTLA4 (MFI)	6473	(5441, 7504)	910	(-549, 2369)	0.221	1882	(423, 3341)	0.011
PD-1 (MFI)	2486	(2069, 2902)	680	(90, 1269)	0.024	-237	(-826, 353)	0.431
TIM3 (MFI)	2	(-51, 56)	53	(-23, 129)	0.169	95	(19, 171)	0.015
LAG-3 (MFI)	969	(907. 1032)	26	(-63, 115)	0.566	-76	(-165, 12)	0.091
**Figure 6C**								
4exh markers (%)	5.2	(4.3, 6.1)	5.4	(4.1, 6.6)	<0.001	6.7	(5.5, 8.0)	<0.001

Analysis done with Stata 15 mixed, REML option.

### PG/VG and nicotine exposure leads to increased metastases *in vivo*

**Figure 2:**
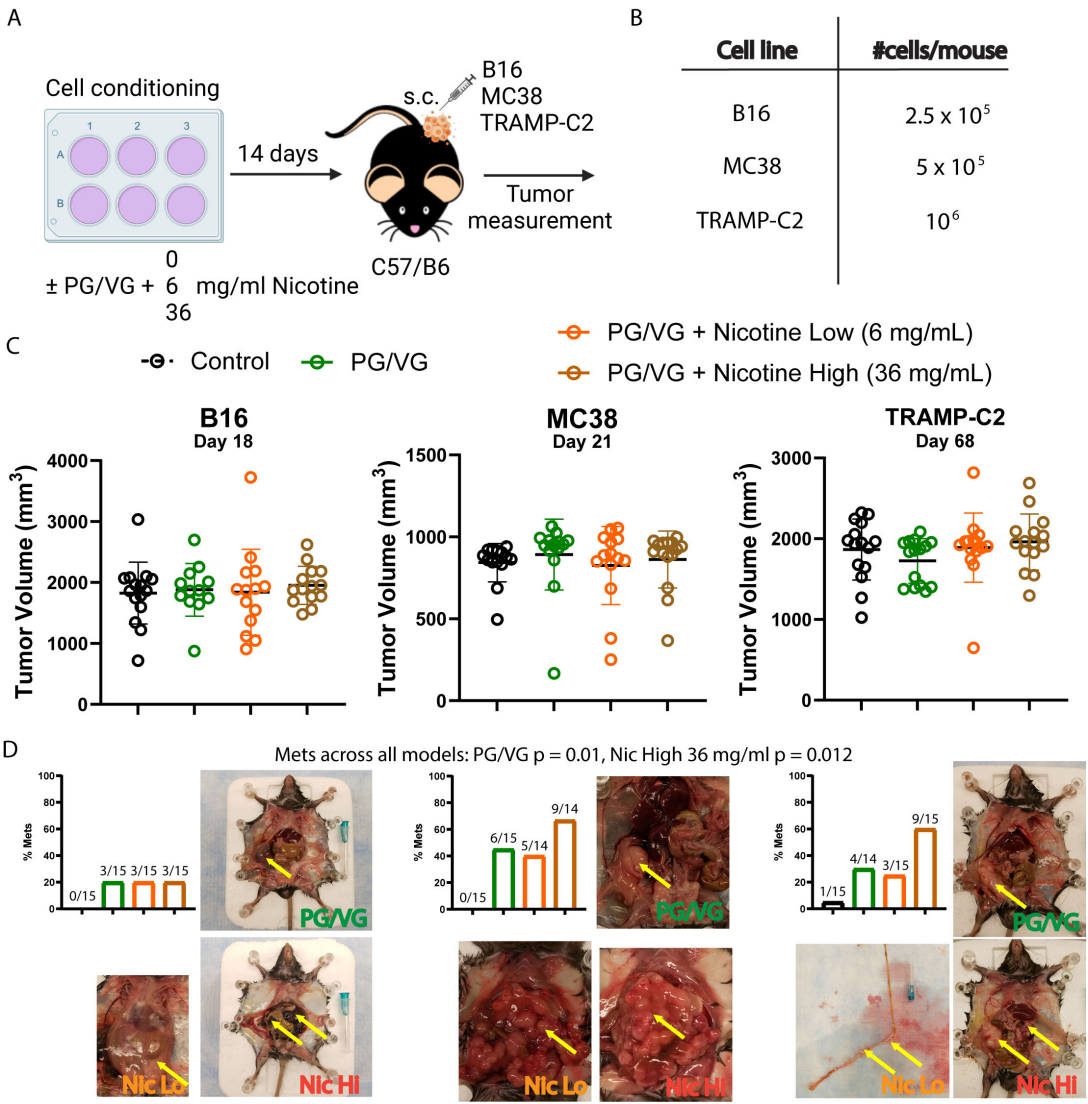
Preconditioning with PG/VG increases metastasis in different subcutaneous tumor models. **(A)** Experimental scheme to test the impact of PG/VG and nicotine on the outcome of subcutaneous tumors. Tumor cells were cultured in the presence of 2.5 μM PG/VG with concentrations of nicotine of 0, 6 and 36 mg/ml for 14 days. Then, preconditioned cells were implanted subcutaneously in the right flanks of wildtype C57/B6 mice (5 mice per experiment, n=3 experiments). Tumors were measured twice weekly and animals were inspected for metastases after euthanization. **(B)** Viable cells implanted per mouse for each tumor model: melanoma B16, colorectal MC38 or prostate TRAMP-C2. **(C)** Endpoint tumor volumes for B16, MC38 and TRAMP-C2 models. Error bars represent standard error mean (SEM). Neither PG/VG nor nicotine affected tumor volume (B16: PG/VG p=0.876, nicotine p=0.601; MC38: PG/VG p=0.740, nicotine p=0.914; TRAMP-C2, PG/VG p=0.500, nicotine p=0.130; **Table 2**). **(D)** Bar plots showing metastasis rates for each subcutaneous tumor model. Logistic regression showed increased odds of metastases associated with exposure to PG/VG (14.9; 95% CI 1.9–116.7; p=0.010) and nicotine (2.9; 1.3–6.9, p=0.012 at 36 mg/mL), controlling for tumor type. Representative pictures with peritoneal metastases are shown for mice exposed to PG/VG with or without nicotine for each tumor model. Control animals (black bars) were not exposed to PG/VG or nicotine. Yellow arrows indicate highly metastasized areas. Metastasis was extremely rare in mice not exposed to PG/VG or nicotine (0/15 in B16 and MC38, 1/15 in TRAMP-C2).

To test the relevance of our findings *in vivo*, we implanted three tumor cell lines (melanoma B16, colorectal MC38, and prostate TRAMP-C2) that had been previously incubated with PG/VG or nicotine into mice subcutaneously and monitored tumor growth (**Figure 2A, B**). Unlike the observed *in vitro* effects on tumor cell migration, there was no observable impact in endpoint primary tumor volumes in any of the models (**Figures 2C**, **Table 2**). Interestingly, PG/VG, but not nicotine, significantly increased tumor growth rate in the B16 melanoma model, consistent with the increased proliferation seen *in vitro*, with bigger differences over time (**Supplementary Figure S2**). We found peritoneal metastases occurring in 20% (6/30, B16), 50% (14/28, MC38) and 40% (12/30, TRAMP-C2) of mice harboring PG/VG+nicotine-exposed tumors, as well as in 20% (3/15, B16), 40% (6/15, MC38) and 28.6% (4/14, TRAMP-C2) for mice harboring PG/VG-exposed tumors, in striking contrast with the absent (0/15, B16 and MC38) or rare metastases (1/15, TRAMP-C2) unexposed tumors (**Figure 2D**). Our statistical analysis showed significant and independent effects of both PG/VG and nicotine on the increased occurrence of metastases with PG/VG having a bigger effect (**Figure 2D**). These findings are consistent with the ability of PG/VG to promote tumor cell invasiveness *in vitro*, but also highlight the effects e-cigarette components can have in tumor dissemination.

**Table 2:** Tumor growth analysis.

Supplementary Figure S2 Tumor growth over time (ln tumor volume, mm^3^)									
	Est.	95% CI	P	Est.	95% CI	P	Est.	95% CI	p
	B16 (melanoma)			MC38 (colorectal)			TRAMP-C2 (prostate)		
Days (per day)	1.86	(1.72, 1.99)	<0.001	0.80	(0.70, 0.89)	<0.001	0.29*	(0.27, 0.31)	<0.001
Days^2^ (per day^2^)	-0.05	(-0.05, -0.04)	<0.001	-0.17	(-0.02, -0.01)	<0.001	-0.005	(-0.005, -0.004)	<0.001
PG/VG [no/yes]	4.22	(2.95, 5.50)	<0.001	-0.58	(-0.78, 0.89)	0.892	0.41	(-0.15, 0.97)	0.156
PG/VG x days (per day)	-0.25	(-0.32, -0.17)	<0.001	-0.01	(-0.05, 0.04)	0.749	-0.02	(-0.04, 0.00)	0.069
Nicotine (0, 6, 36 mg/ml) (per mg/ml)	-0.02	(-0.06, 0.01)	0.169	0.00	(-0.02, 0.03)	0.749	-0.01	(-0.03, 0.01)	0.280
Nicotine x days (per (mg/ml)/day)	0.00	(-0.002, 0.004)	0.093	0.00	(-0.001, 0.001)	0.850	0.00	(-0.000, 0.001)	0.065
Constant (Control) (%)	-10.99	(-12.32, -9.66)	0.001	-2.66	(-3.54, -1.77)	<0.001	2.95	(2.51, 3.40)	<0.001
Figure 2C. Tumor volume at end of study (not transformed)									
PG/VG [no/yes]	26.7	(-308.6, 361.9)	0.876	20.8	(-102.0, 143.6)	0.740	-79.5	(-310.6, 151.6)	0.500
Nicotine (0, 6, 36 mg/mL) (per mg/mL)	2.66	(-7.30, 12.61)	0.601	-0.192	(-3.671, 2.287)	0.914	5.18	(-1.52, 11.88)	0.130
Figure 6D. Tumor volume at end of study (not transformed)									
PG/VG plus 36 mg/mL nicotine (Y/N)				47.4	(-117.1, 211.9)	0.572			
aCTLA4†				-1569	(-1734, -1405)	<0.001			

Analysis conducted with Stata 15 mixed, REML, unstructured covariance options, allowing intercepts and slopes to vary by mouse, i.e., days nested in mice.

Zero values dropped because ln(0) is undefined.

*(Days-40) used as days variable because TRAMP-C2 takes around 40–45 days to become palpable.

†We also tested a model that had the PG/VG+nicotine x aCTLA4 interaction. The interaction was not statistically significant (p=0.934), so it is not included in the analysis presented in this table.

### Tumor cell exposure to PG/VG leads to more aggressive systemic tumors without an additive effect from nicotine

**Figure 3:**
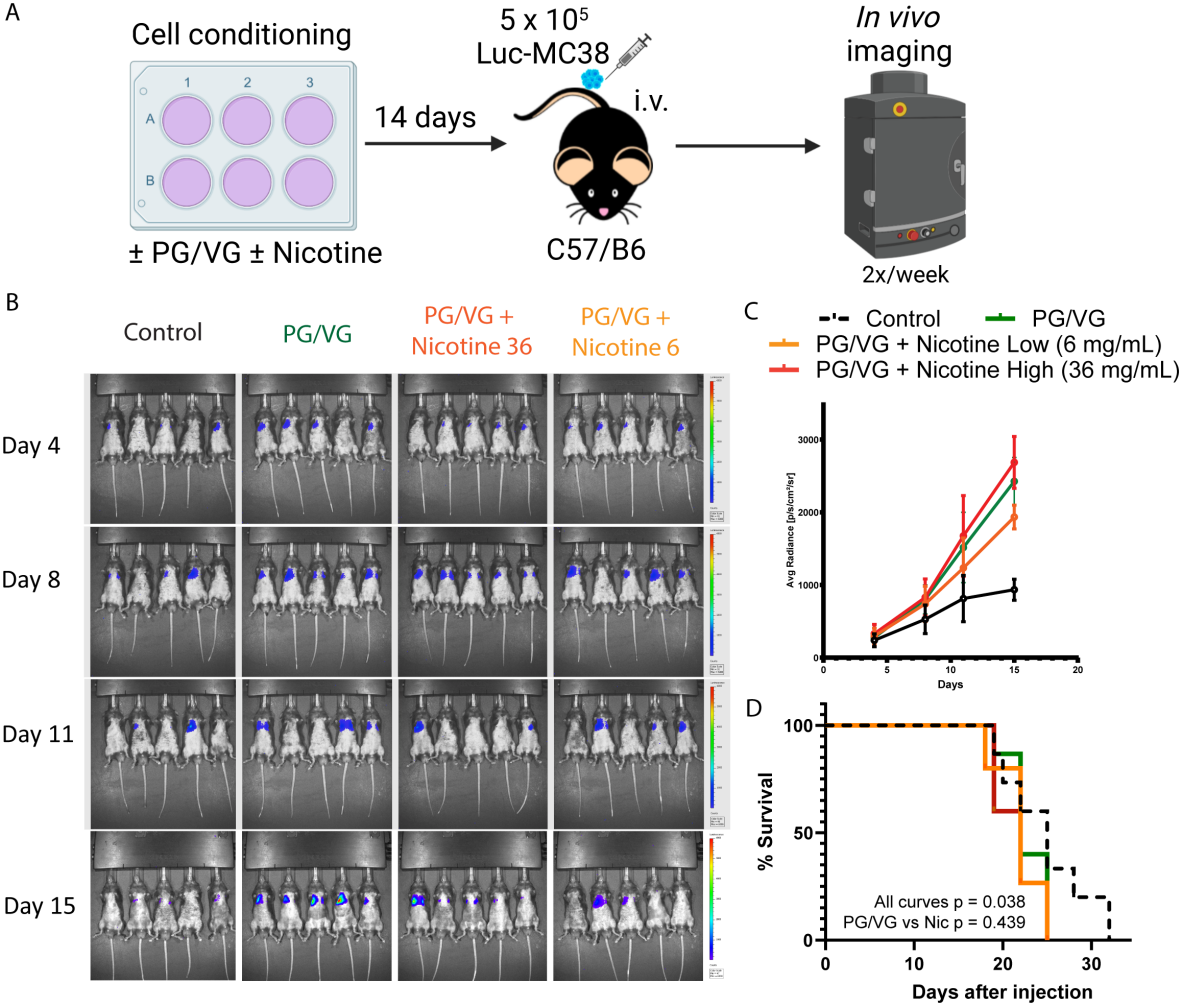
Accelerated tumor progression upon PG/VG preconditioning. **(A)** Experimental scheme to test the impact of PG/VG and nicotine in a model of disseminated cancer. Luciferase-expressing colorectal cancer MC38 (Luc-MC38) cells were cultured in the presence of PG/VG with concentrations of nicotine of 0, 6 and 36 mg/mL for 14 days. Then, 5 x 10^5^ preconditioned Luc-MC38 cells were injected intravenously in the tail veins of wildtype C57/B6 mice (n=5 per experiment, n=3 experiments). Tumor bioluminescence was monitored twice weekly for 15 days. **(B)** Representative whole body bioluminescence images on days 4, 8, 11 and 15 post-implantation is shown. **(C)** Time-course measurement of tumor bioluminescence expressed as Average Radiance for mice involved in experiments described in **(A)**. Error bars represent SEM. **(D)** Kaplan-Meier survival curves for the 3 experiments. There were significant differences between all four curves (p=0.038, by log rank test). There was no significant difference between the PG/VG, PG/VG plus low nicotine, and PG/VG plus high nicotine curves (p=0.439).

We utilized the luciferase-expressing MC38 (Luc-MC38) colorectal cancer tumor cells to longitudinally monitor tumor progression by bioluminescence imaging (**Figure 3A**). The injected tumor cells deposit in the lungs and form tumoral niches, constituting a model for lung metastasis. By day 15 after tumor cell injection, PG/VG-exposed tumor cells showed significantly increased luminescence radiance compared to the control group, evidencing more tumor burden. The addition of nicotine did not show a statistically additive effect over PG/VG (**Figures 3B, C** and **Supplementary Figure S3**). Exposure to PG/VG was also significantly associated with decreased survival rates compared to unexposed mice, again with no additive effect of nicotine (**Figure 3D**). These results reinforce our previous findings involving the enhanced invasiveness of cells exposed to PG/VG.

### Vaped PG/VG and nicotine lead to increased tumor progression

**Figure 4:**
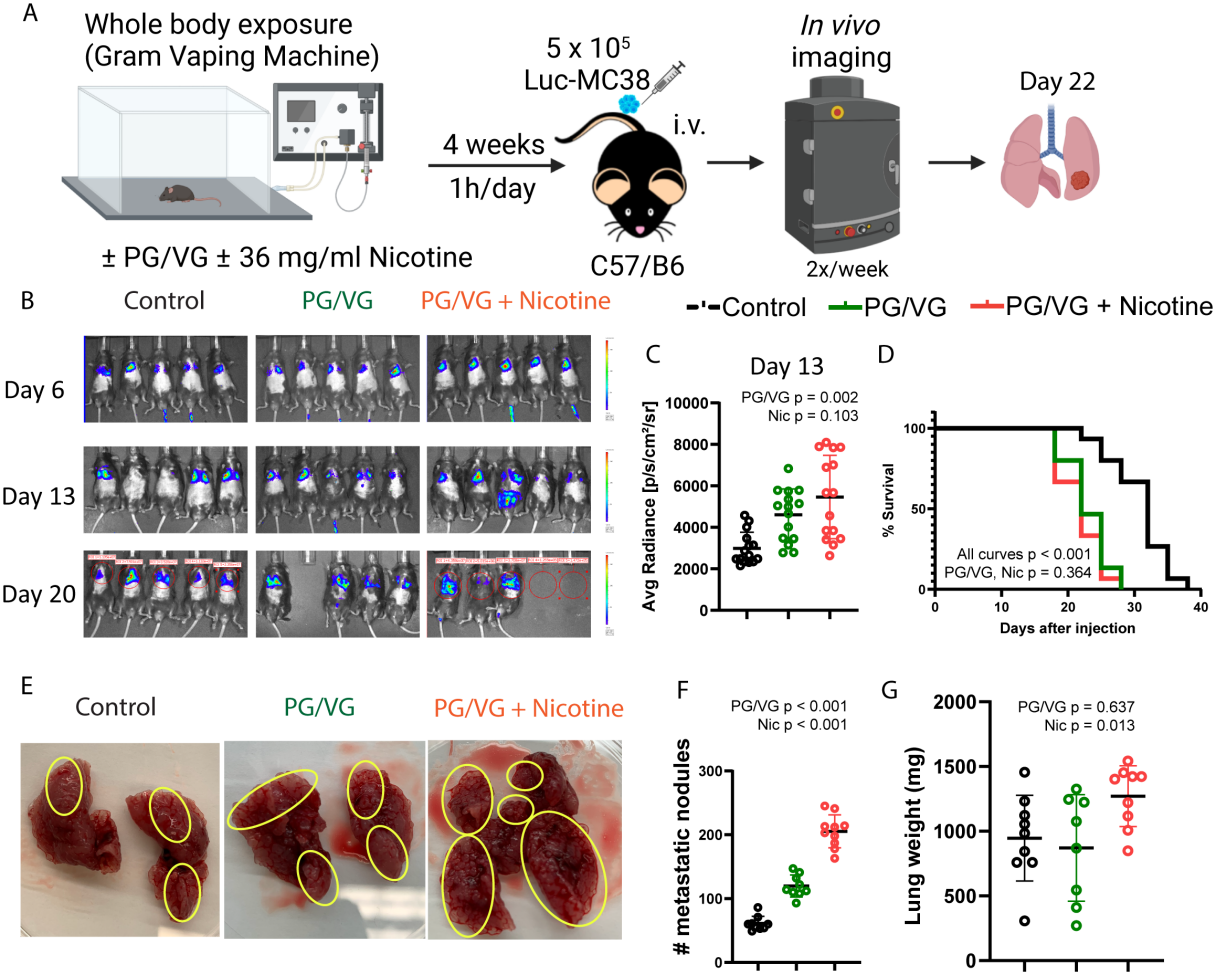
Whole body exposure of mice e-cigarette aerosol leads accelerated tumor growth and more aggressive metastasis. **(A)** Experimental scheme for whole body exposure experiments. Mice were exposed to e-cigarette aerosol with 2.5 µM PG/VG and 0 or 36 mg/ml nicotine for 1h daily for 4 weeks. Then, mice (n=5 per experiment, n=3 experiments) were challenged intravenously with 5 x 10^5^ Luc-MC38 cells. Tumor bioluminescence was monitored twice weekly. Lungs from three mice per experimental condition were harvested on day 22 post-implantation, weighed, inspected for metastasis burden and immunophenotyped by flow cytometry. **(B)** Representative whole body bioluminescence images on days 6, 13 and 20 post-implantation is shown. **(C)** Tumor bioluminescence on day 13 after implantation expressed as average photon radiance was significantly increased by PG/VG (p=0.002; **Table 1**) but not nicotine (p=0.103). **(D)** Kaplan-Meier survival curves from whole body experiments described in **(A)**. There were significant differences between all three curves (p,0.001, by log rank test). There was no significant difference between the PG/VG and PG/VG plus nicotine curves (p=0.364), suggesting that the survival was reduced by exposure to PG/VG with no additional effect of nicotine effect. **(E)** Representative images from surgically extracted lungs 22 days after tumor cell injection. Yellow circles indicate areas with metastatic MC38 nodes. **(F)** Metastatic node count from day 22 lungs increased significantly with exposure to PG/VG (p<0.001; **Table 1**), with a further increase when nicotine was added (p<0.001). **(G)** Lung weight on day 22 after tumor injection was not affected by PG/VG (p=0.637; **Table 1**), but increased with the addition of nicotine (p=0.013).

We performed whole-body exposure experiments in which mice inhaled aerosolized e-cigarette liquids 1 hour daily for 4 weeks. Following this exposure, we injected Luc-MC38 colorectal cancer cells intravenously (**Figure 4A**). In line with the preconditioning experiments, exposure to inhaled PG/VG led to a higher tumor burden as early as day 13 post tumor implantation. Nicotine did not modulate this effect (**Figures 4B, C** and **Supplementary Figure S4**, **Table 1**). Again, this increase in tumor aggressiveness translated into significantly diminished survival rates for mice exposed to PG/VG that was not affected by adding nicotine, with first deaths occurring as early as day 18 post implantation (**Figure 4D**). We harvested the lungs from mice for downstream analysis in each of these studies at day 22 after implantation for further characterization. We found significantly increased metastatic nodule counts in lungs from PG/VG- and nicotine-exposed mice, reaching over 200 nodes in a pair of lungs (**Figures 4E, F**). Nicotine exposure also led to significantly increased lung weight, but PG/VG alone had no apparent effect (**Figure 4G** and **Supplementary Figure S4**, **Table 1**).

### Vaped PG/VG and nicotine lead to immunosuppression

**Figure 5:**
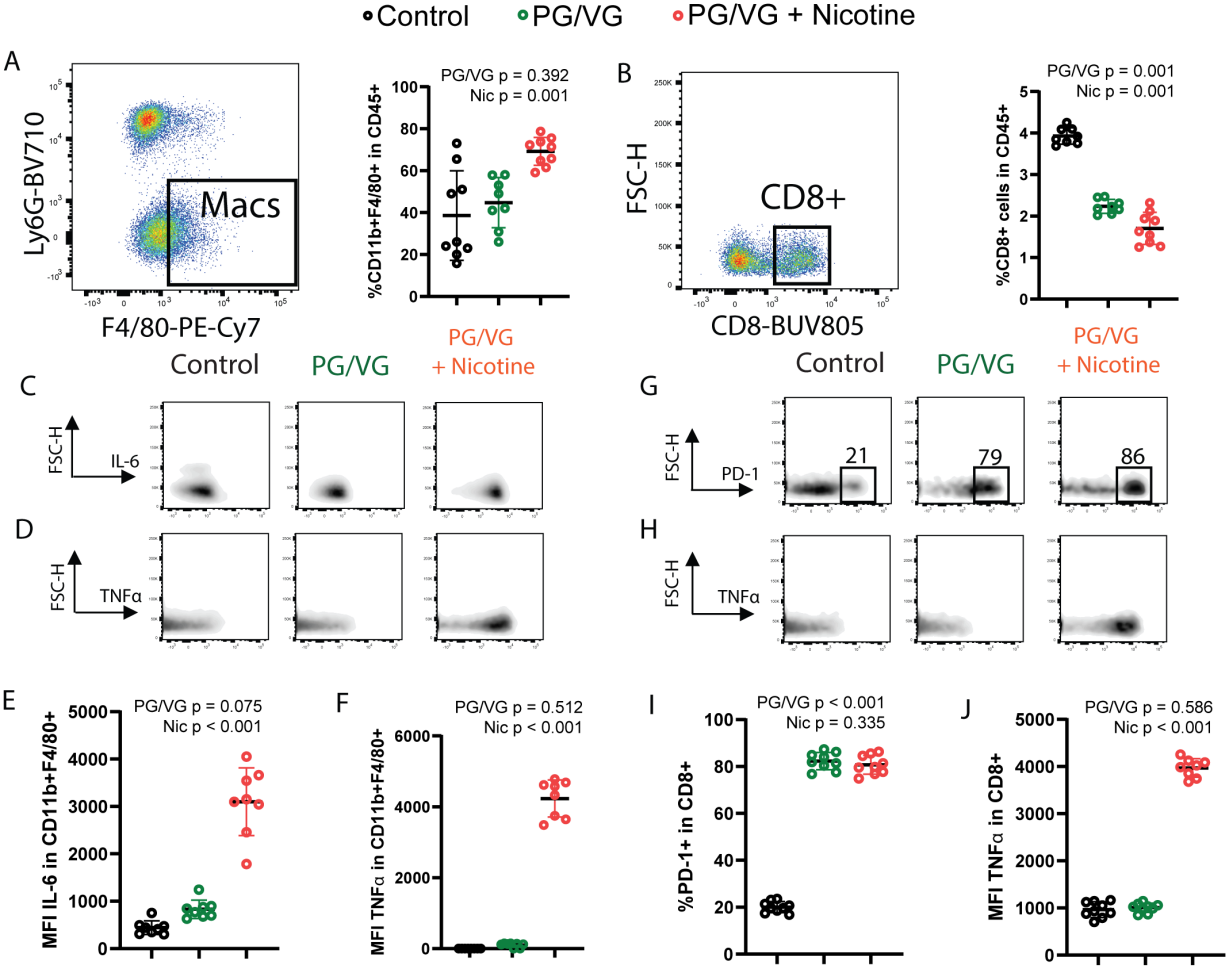
Whole body exposure of mice e-cigarette aerosol leads to increased myeloid and lymphoid immunosuppression in the tumor microenvironment. **(A)** Left, representative flow cytometry pseudocolor dot plot showing gating on lung-infiltrating macrophages from animals exposed to PG/VG with or without 36 mg/ml nicotine as described in **Figure 4A**. Right, lung-infiltrating macrophage frequency within gated live CD45+ immune cells was not affected by PG/VG (p=0.392; **Table 1**) but increased with the addition of nicotine (p=0.001). **(B)** Left, representative flow cytometry pseudocolor dot plot showing gating on lung-infiltrating CD8+ T cells. Right, lung-infiltrating CD8+ T cell frequency within gated live CD45+ immune cells fell significantly with exposure to PG/VG (p<0.001; **Table 1**) and about one-third more with the addition of nicotine (p<0.001). **(C)** Representative flow cytometry density plots showing IL-6 expression in gated lung-infiltrating macrophages. **(D)** Representative flow cytometry density plots showing TNFα expression in gated lung-infiltrating macrophages. **(E)** Mean fluorescence intensity of IL-6 in gated lung macrophages was not affected significantly by PG/VG (p=0.075; **Table 1**) but increased significantly with the addition of nicotine (p<0.001). **(F)** Mean fluorescence intensity of TNFα in gated lung macrophages was not affected significantly by PG/VG (p=0.512; **Table 1**) but increased significantly with the addition of nicotine (p<0.001). **(G)** Representative flow cytometry density plots showing PD-1 expression in gated lung-infiltrating CD8+ T cells. **(H)** Representative flow cytometry density plots showing TNFα expression in gated lung- infiltrating CD8+ T cells. **(I)** Percentage PD-1+ cells in gated lung CD8+ T cells was significantly higher in the presence of PG/VG (p<0.001; **Table 1**) but not affected by addition of nicotine (p=0.335). **(J)** Mean fluorescence intensity of TNFα in gated lung CD8+ T cells was not significantly different in the presence of PG/VG (p=0.586; **Table 1**) but significantly increased with the addition of nicotine (p<0.001).

Because anti-tumor immunity is important in suppressing tumor progression, we performed flow cytometry on dissociated cells from the lungs at day 22 to assess how exposure modulates the tumor immune microenvironment. Lungs from nicotine-exposed mice possessed significantly increased macrophage infiltration, representing up to 70% of all CD45+ immune cells of (**Figure 5A** and **Supplementary Figure S5**, **Table 1**). Exposure to PG/VG aerosol also led to significantly decreased frequency of CD8+ T cells, which was minimally worsened with the addition of nicotine (**Figure 5B** and **Supplementary Figure S5**). Exposure to PG/VG aerosol also induced minimal IL-6 production in lung-infiltrating macrophages. We did not observe significant differences in macrophage TGFβ across groups (**Supplementary Figure S5**). However, lung-infiltrating macrophages from nicotine-exposed mice expressed significantly higher levels of IL-6 and TNFα (**Figures 5C-F** and **Supplementary Figure S5**), indicating that nicotine helps to drive inflammation within the TME. We also observed significantly higher TNFα levels in lung-infiltrating CD8+ T cells from nicotine-exposed mice. Combined with lack of proliferation (Ki67-) or reduced expression of canonical markers for antitumoral responses such as interferon gamma (IFNγ) or cytolytic Perforin (PRF1) in these CD8+ T cells, this suggests impaired antitumoral T cell fitness and contribution to protumoral inflammation (**Supplementary Figure S5**). Strikingly, exposure to PG/VG alone or in combination with nicotine led to significantly higher frequencies of exhausted PD-1-expressing, Ki67-negative, CD8+ T cells with impaired cytotoxicity (**Figures 5G-J** and **Supplementary Figure S5**). Notably, CD4+FoxP3+ T regulatory (Treg) frequencies did not vary significantly between groups, although an increasing trend was observed upon nicotine exposure (**Supplementary Figure S5**). Together, these findings indicate that PG/VG alone elicits immunosuppression, but when combined with nicotine, induces inflammation that may enhance tumor dissemination.

### Tumors exposed to PG/VG and nicotine are responsive to immune checkpoint inhibition

**Figure 6:**
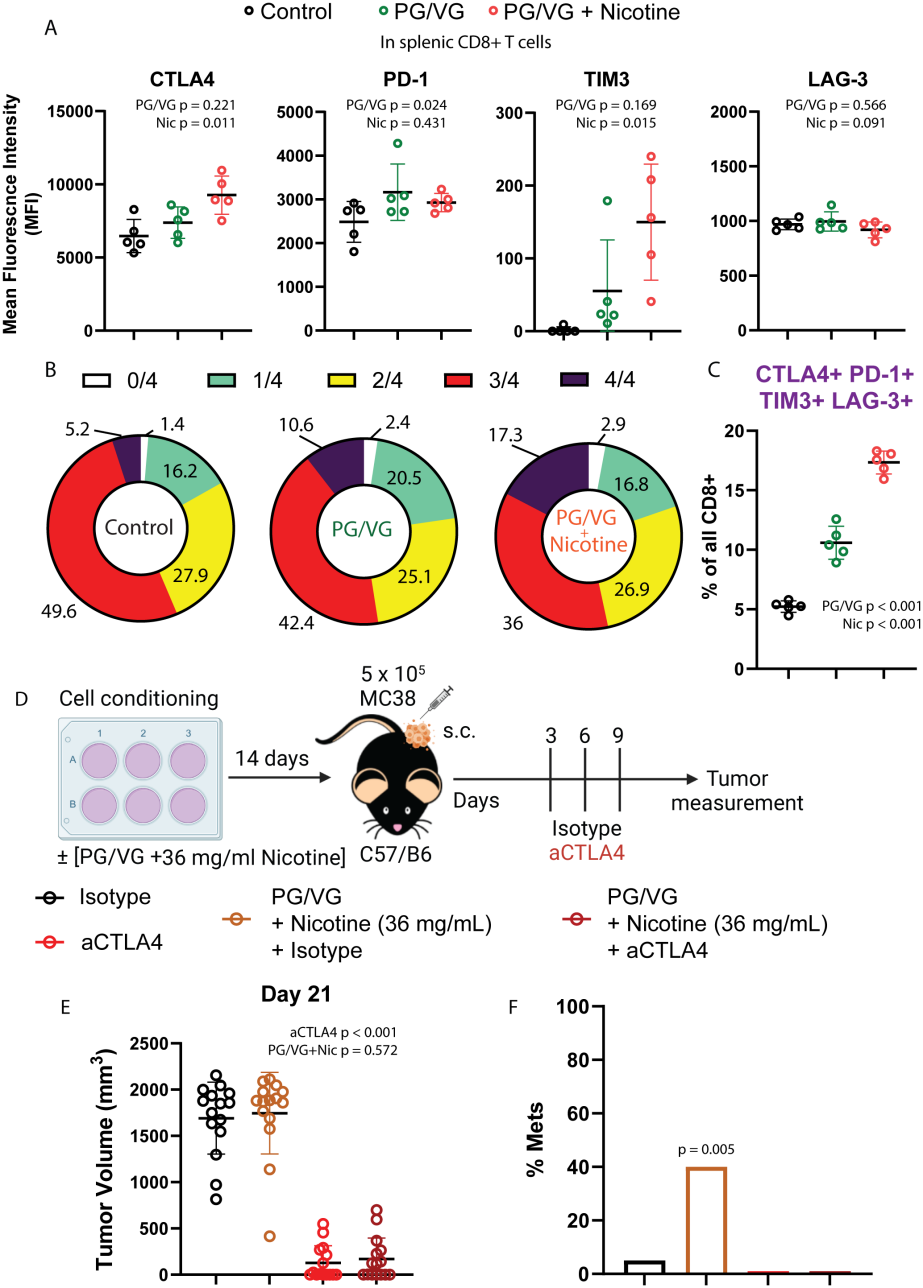
Tumors exposed to proinflammatory e-cigarette components are responsive to immune checkpoint blockade. **(A)** Mean fluorescence intensity of T cell immune checkpoints CTLA4, PD-1, TIM3 and LAG-3 on splenic CD8+ T cells from mice exposed to PG/VG with or without 36 ml/mg nicotine as described in **Figure 4A**. CTLA4 and TIM3 were significantly elevated only in the presence of nicotine (p=0.011, p=0.015 respectively). PD-1 was significantly elevated in the presence of PG/VG (p=0.024) with no nicotine effect (p=0.431). No significant differences were found for LAG-3 expression. Summarized results can be found in **Table 1**. **(B)** The frequency of immune checkpoint co-expression (none (0), any given one (1), any combination of 2 (2), any combination of 3 (3) or all markers (4)) is shown in splenic CD8+ T cells as assessed by flow cytometry Boolean gating. **(C)** Percentage of quadruple-positive CTLA4+TIM3+LAG-3+PD-1+ splenic CD8+ T cells. Significantly elevated quadruple-positive cells were found in the presence PG/VG (p<0.001, **Table 1**) with a significant additive nicotine effect (p<0.001). **(D)** Experimental scheme to test the effect of immune checkpoint inhibition in e-cigarette preconditioned subcutaneous tumors. After preconditioning MC38 cells in the presence of 2.5 μM PG/VG with or without 36 mg/ml nicotine for 14 days, mice (n=5 per experiment) were implanted with tumors subcutaneously. Starting 3 days after implantation., mice were treated with intraperitoneal anti-CTLA4 (aCTLA4) or the relevant isotype control (IgG2k/a) on days 3, 6 and 9 after implantation. **(E)** Endpoint tumor volumes. Error bars represent standard error mean (SEM). Treatment with anti-CTLA4 showed a significant effect on tumor volume (p<0.001; **Table 2**) but not observed for PG/VG+nicotine (p=0.572). No significant interaction between anti-CTLA4 treatment and presence of PG/VG+nicotine was observed (p=0.934). **(F)** Metastasis rates: 6/15 (40%) animals with PG/VG-preconditioned MC38 tumors showed peritoneal metastases, which were absent (0/15) in both anti-CTLA4-treated groups and nearly absent (1/15) in control mice. There was no significant difference in metastases among these three groups (p=0.762 by Fisher Exact Test (p=0.762) and a significant increase in the PG/VG+nicotine mice compared to the others (p=0.005 by Fisher Exact Test).

Given the observed impact of PG/VG and nicotine on the immune landscape of tumors, we analyzed the circulating T cell compartment for expression immune checkpoints to assess for systemic immune effects. Despite not showing differences in frequencies across groups (**Supplementary Figure S6**), splenic CD8+ T cells from mice exposed to PG/VG and nicotine expressed significantly higher levels of the immune checkpoints CTLA4, PD-1 and TIM3, while maintained comparable levels of LAG-3 to unexposed mice (**Figure 6A**). To assess the degree of exhaustion in these cells by co-expression of multiple immune checkpoints, we applied Boolean gating and found that 10.6% of PG/VG-exposed splenic CD8+ T cells showed co-expression of all 4 immune checkpoints, significantly doubling the levels on control CD8+ T cells (5.22%). Moreover, the presence of nicotine showed a significant additive effect, elevating circulating CTLA4+ PD-1+ TIM3+LAG-3+ CD8+ T cells to 17.34% within the CD8 compartment (**Figures 6B, C**), in line with the higher levels of exhaustion detected in the tumors.

To examine whether these immune checkpoint are limiting antitumor responses, we tested the sensitivity of e-cigarette-exposed subcutaneous tumors to immunotherapy in the MC38 model (25) by treating mice with the immune checkpoint inhibitor anti-CTLA4 (**Figure 6D**). In this model, anti-CTLA4 therapy prevented tumor growth independently of the presence of PG/VG + 36 mg/mL nicotine (**Figure 6E** and **Supplementary Figure S6**, **Table 2**). Notably, anti-CTLA4 treatment completely prevented the appearance of peritoneal metastases in e-cigarette-exposed animals (**Figure 6F**). These findings show that CTLA-4 represents a functional checkpoint in this setting.

## Discussion

Even though neither PG/VG nor nicotine are carcinogens (i.e., tumor initiators), we found both *in vitro* and *in vivo*, that exposure to these compounds promotes tumor growth and occurrence of metastasis across multiple preclinical tumor models. PG/VG stimulates these processes in a dose-dependent manner and nicotine often, but not always, amplifies these effects.

Our results are consistent with the one earlier study examining the effect of e-cigarette exposure on tumor growth and metastasis, which examined the effect on breast cancer tumor grown and lung metastasis in mice (19). Like us, Pham et al. used a mixture of 50:50 PG/VG and nicotine (24 mg/mL) and identified the protumoral role of tumor-infiltrating suppressive macrophages upon their CCR5:CCL5 crosstalk with tumor cells, enhanced by exposure to a mixture of PG/VG and nicotine. They did not separate the effects of PG/VG and nicotine and did not characterize the lymphoid compartment infiltrating the breast and metastatic tumor models. The present study validates the proinflammatory macrophage infiltration described by Pham and colleagues as well as the other reports showing the association between e-cigarettes and IL-6 upregulation in humans and mice (20, 23, 26, 27). This study goes beyond the existing literature to identify important pro-metastatic roles for PG/VG and nicotine, as well as their involvement in immunosuppression both locally within the tumor and systemically in circulating lymphocytes.

PG/VG is not inert. Rather, PG/VG contributes to *in vitro* tumor cell invasion, as well as *in vivo* metastasis and systemic tumor aggressiveness. Biological effects of PG/VG have already been described including upregulation of extracellular matrix components (20), increased pulmonary and airway mucus (28–30), increased oxidation and DNA damage (31, 32), and metabolic alterations that compromise airway epithelial barriers (33). We also found that PG/VG exposure induced a reduction in CD8 T cells, a cell type critical for mediating anti-tumor immunity, providing a potential mechanism for enhanced tumor growth. This result is consistent with Sciezska and colleagues’ finding of impaired lung immunosurveillance in the form of decreased CD8+ T cells in lungs from animals exposed to PG/VG (34). We also found that CD8+ infiltrating PG/VG-exposed tumors showed increased levels of the immune checkpoints, further hampering effective antitumoral responses and clearance of cancerous cells. Together, these data support the notion that PG/VG can render exposed hosts more vulnerable to immunological challenges, such as viral infections (23, 35) or the development or dissemination of tumors, in addition to promoting tumor-intrinsic mechanisms leading to changes in tumor cell survival and invasion. Future studies diving into the molecular aspects of such changes could help better understand the formation of premetastatic niches.

While nicotine does not contribute to tumor initiation in our studies, it can make existing tumors worse. Nicotine affects several biological pathways, including cancer-relevant ones (10–12, 36–38). In our study, nicotine’s presence *in vivo* led to immune-intrinsic pro-tumoral changes, ranging from increasing macrophage- and T cell-induced immunosuppressive inflammation within the tumor microenvironment, decreasing CD8+ T cell infiltration and proliferation or increasing T cell exhaustion. Our immunological findings are consistent with previous immunoinhibitory roles reported for e-cigarette nicotine *in vitro (* 14) and *in vivo (* 15, 23, 34). One of such effects might be the observed upregulation of TNFα in immunosuppressive, tumor-infiltrating macrophages, previously reported in the context of chronic e-cigarette exposure (39, 40). The lack of differences in TGFβ might be explained by its more critical role in forming the metastatic niche and its expression from lung epithelial cells rather than macrophages (30). On the other hand, impaired anti-tumoral CD8+ T cell responses from PBMCs derived from smoker subjects have been shown in humanized tumor xenografts (15); while we observed a modest effect in Treg infiltration, we demonstrated that induction of inhibitory immune checkpoints such as PD-1, TIM3, LAG-3 or CTLA-4 on T cells occurs in syngeneic models upon e-cigarette exposure. Furthermore, we demonstrate that expression of these checkpoints is consequential, since treatment with anti-CTLA-4 antibodies can induce tumor regression in PG/VG plus nicotine-exposed animals. While Madison and colleagues found no correlation between the presence of nicotine and innate immune disruption caused by e-cigarettes (41), our data strongly suggest nicotine can impair adaptive immune responses.

These findings suggest the need to study cell-cell interactions to identify additional specific biological, metabolic pathways or critical infiltrating cell populations both to understand mechanisms of action and identify potential therapeutic or preventive interventions. Given the reported relevance for both lymphoid (14, 15, 21, 23, 34) and myeloid (19, 42) populations in antitumor responses in the presence of e-cigarette components, reinforced by the findings in this study, more preclinical studies involving exposure to PG/VG, nicotine and other e-cigarette components with targeted depletion of immune populations could be very informative to identify key targetable cell subsets, pathways or molecules.

This work is subject to several limitations. Firstly, the exclusive use of male mice in our experiments does not allow assessment of gender-based differences in the effect of e-cigarette exposure. Complementary studies in female mice will ascertain the extent of such differences. Because it is not possible to aerosolize nicotine without a humectant (in this case PG/VG), in most of the experiments we exposed the cells or animals to PG/VG or PG/VG plus nicotine. This arrangement allows us to draw conclusions about PG/VG and the marginal effect of adding nicotine, but limits conclusions about nicotine alone. The PG: VG ratio in commercially available e-cigarettes is not regulated and varies widely (43–45). We studied a previously reported relevant mixture of 1:1 PG/VG and nicotine, not whole aerosol generated by commercial e-cigarettes, which also includes flavoring and other components, as well as a range of PG: VG ratios. Future work could try to disentangle the roles of VG and PG separately. Finally, while we report the response of mouse cancers to relatively short exposures to PG/VG and nicotine, the specific relevance to chronic human exposure to these e-cigarette components, together with other components of e-cigarettes such as flavors, remains to be determined.

In 2012, the Food and Drug Administration established a list of Harmful and Potentially Harmful Constituents in Tobacco Products and Tobacco Smoke (46) (HPHC), which is dominated by toxicants in cigarette smoke. The HPHC list provides key metrics for assessing the harmfulness of tobacco products. In 2019 the FDA proposed adding 19 compounds to the HPHC list, including propylene glycol and glycerol, to reflect compounds in e-cigarettes (47). While e-cigarette components may induce lower carcinogenesis (although there is little data beyond lower biomarkers of exposure), there are many indirect effects that must be considered when assessing their health impact, including promotion of metastasis or immunosuppressive infiltration documented here. The results presented in this study underscore the consideration of propylene glycol as a harmful component given its widespread use in e-cigarettes as well as heated tobacco products (48).

Our results demonstrate new potential risks associated with e-cigarettes. Future assessments of the safely of e-cigarettes should include not only incidence of cancer, but also acceleration of cancers caused by other agents.

## Data Availability

The original contributions presented in the study are included in the article/**Supplementary Material**. Further inquiries can be directed to the corresponding author.

## Supplementary material

Supplementary Figure 1 E-cigarette chemicals promote tumor cell migration *in vitro*. **(A)** Percent of scratch area covered by MC38 cells at 0, 4, 12 and 24h after insert removal in cell migration assays. P=PG/VG at 2.5 µM. **(B)** Viable cells on days 3, 5 and 7 after cell seeding.

Supplementary Figure 2 PG/VG or nicotine impact primary growth for B16 but not M38 and TRAMP-C2 tumors. **(A)** Tumor growth analysis over time for experiments described in **Figure 5**. At any given time, tumor volume is larger in B16 (melanoma) tumors in animals exposed to PG/VG (ln(tumor volume) increased by 4.22 (95%CI 2.95, 5.50), p<0.001; **Table 2**), with a slower rate of grown (ln(tumor volume)/day -0.25 (-0.32, -0.17), p<0.001). Nicotine did not significantly affect tumor volume (p=0.169 for offset, p=0.093 for change in growth rate). Neither PG/VG nor nicotine was significantly associated with changes in tumor growth for MC38 (colorectal) or TRAMP-C2 (prostate) tumors. Lines represent fit curves. Error bars represent SEM. **(B)** Raw tumor volume measurements for subcutaneous B16, MC38 and TRAMP-C2 implantation experiments described in **Figure 5A**. Error bars represent SEM.

Supplementary Figure 3 PG/VG tumor cell preconditioning induces more aggressive Luc-MC38 tumors. **(A)** Tumor bioluminescence on day 15 after implantation expressed as average photon radiance. Tumor bioluminescence was significantly increased in cells preconditioned with PG/VG (p<.001; **Table 1**). Adding nicotine had no additional effect (p=0.187).

Supplementary Figure 4 Whole body exposure of mice e-cigarette aerosol leads accelerated tumor growth and more aggressive metastasis. **(A)** Whole body exposure machine set up for experiments described in **Figure 2**. Briefly, vaped e-cigarette liquids were injected into an exposure chamber containing live mice continuously for 1h, 5 days a week. **(B)** Time-course measurement of tumor bioluminescence expressed as Average Radiance for mice involved in experiments described in **Figure 2**. Error bars represent SEM. **(C)** Representative pictures of harvested lungs from unchallenged mice (left) or e-cigarette-exposed mice (right).

Supplementary Figure 5 Whole body exposure of mice e-cigarette aerosol leads to increased myeloid and lymphoid immunosuppression in the tumor microenvironment. **(A)** Flow cytometry gating strategy used for phenotyping of Luc-MC38 tumor immune infiltrates harvested from lungs of mice involved in experiments described in **Figure 2**. **(B)** Left, offset histograms representing normalized modal frequencies for markers Ki67, IFNγ and PRF1 in gated tumor-infiltrating CD8+ T cells. Right, percentages of Ki67+, IFNγ+ and PRF1+ T cells within the CD8+ compartment. **(C)** Mean fluorescence intensity of TGFβ in gated lung macrophages was not affected significantly (p=0.5257). **(D)** Percentage CD4+FoxP3+ cells in gated CD45+ cells. No significant differences were observed (p=0.1597).

Supplementary Figure 6 Tumors exposed to proinflammatory e-cigarette components are responsive to immune checkpoint blockade. **(A)** Splenic CD8+ T cell frequencies within gated live CD45+ immune cells in mice involved in whole body exposure experiments. **(B)** Representative flow cytometry histograms showing normalized modal frequencies for CTLA4, PD-1, TIM3 and LAG-3 expression in gated splenic CD8+ T cells. **(C)** Tumor volume curves for n=3 experiments described in **Figure 4**. Red arrows indicate Isotype control or anti-CTLA4 treatment. Black lines represent fit curves. Error bars represent SEM.

Supplementary Table 1 Antibodies used for flow cytometry.

Supplementary Table 2 Source data for **Figures 1**–**6**.

Supplementary Table 3 Occurrence of metastases in **Figure 2D**.

Supplementary Table 4 Tumor growth curves in **Figure S2**.
