# Supplementary figures and images for: E-cigarette exposure disrupts antitumor immunity and promotes metastasis
Source: Front Immunol. 2024 Aug 16;15:1444020. doi: 10.3389/fimmu.2024.1444020 (PMC11365074; doi:10.3389/fimmu.2024.1444020)

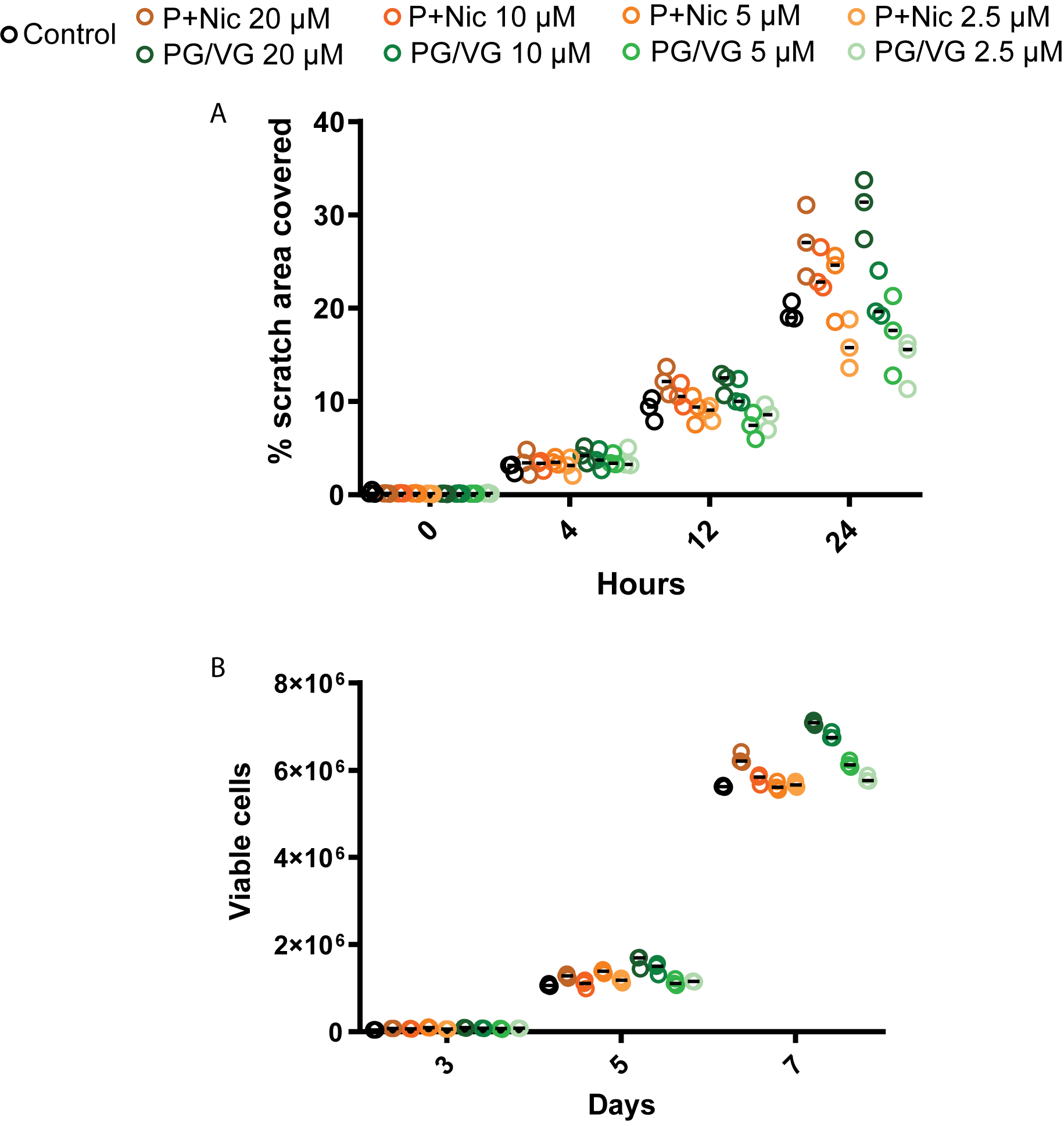

Supplement: Supplementary Figure 1 — E-cigarette chemicals promote tumor cell migration in vitro. (A) Percent of scratch area covered by MC38 cells at 0, 4, 12 and 24h after insert removal in cell migration assays. P=PG/VG at 2.5 µM. (B) Viable cells on days 3, 5 and 7 after cell seeding. [file Image1.tif]

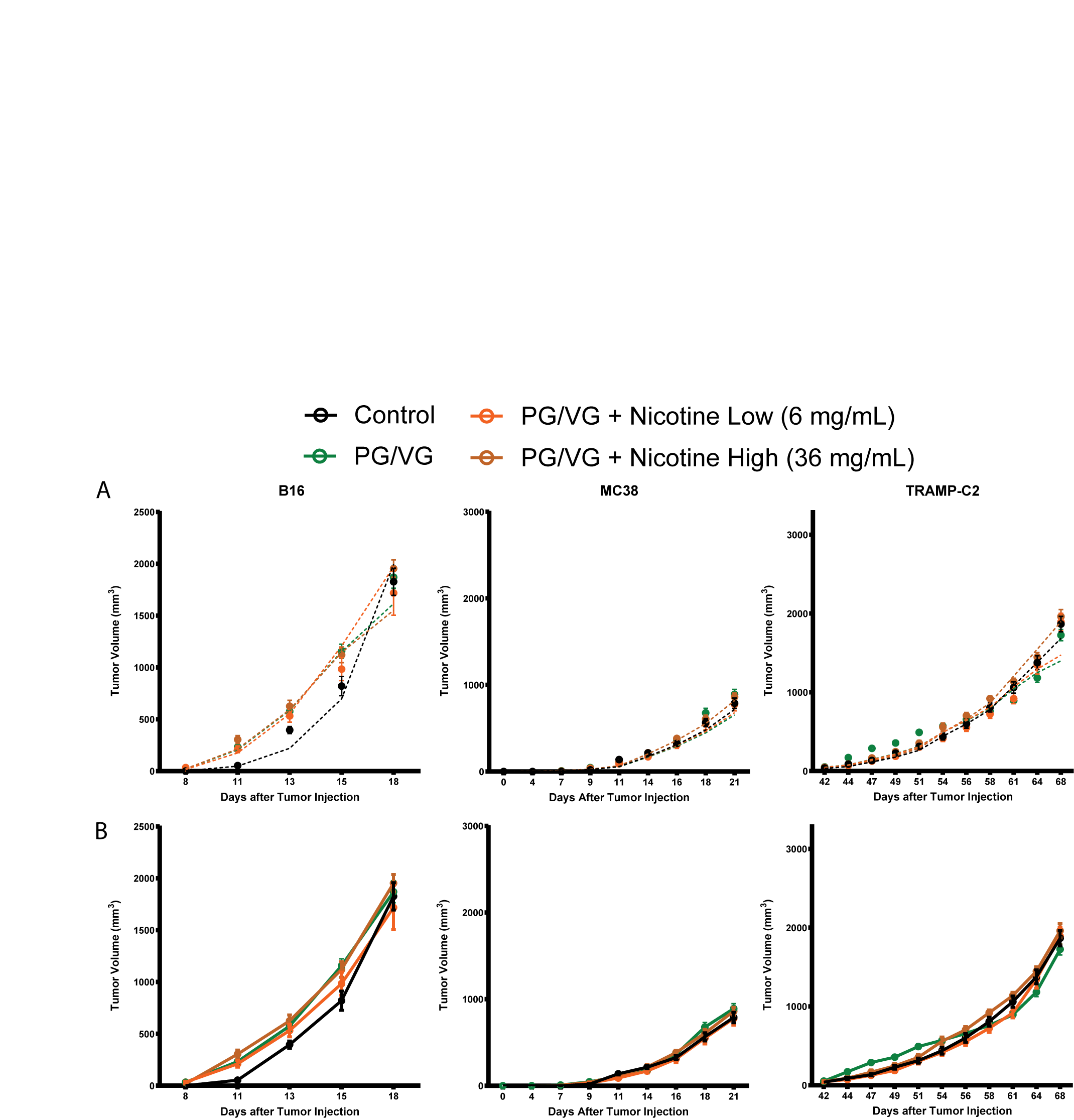

Supplement: Supplementary Figure 2 — PG/VG or nicotine impact primary growth for B16 but not M38 and TRAMP-C2 tumors. (A) Tumor growth analysis over time for experiments described in Figure 5 . At any given time, tumor volume is larger in B16 (melanoma) tumors in animals exposed to PG/VG (ln(tumor volume) increased by 4.22 (95%CI 2.95, 5.50), p<0.001; Table 2 ), with a slower rate of grown (ln(tumor volume)/day -0.25 (-0.32, -0.17), p<0.001). Nicotine did not significantly affect tumor volume (p=0.169 for offset, p=0.093 for change in growth rate). Neither PG/VG nor nicotine was significantly associated with changes in tumor growth for MC38 (colorectal) or TRAMP-C2 (prostate) tumors. Lines represent fit curves. Error bars represent SEM. (B) Raw tumor volume measurements for subcutaneous B16, MC38 and TRAMP-C2 implantation experiments described in Figure 5A . Error bars represent SEM. [file Image2.tif]

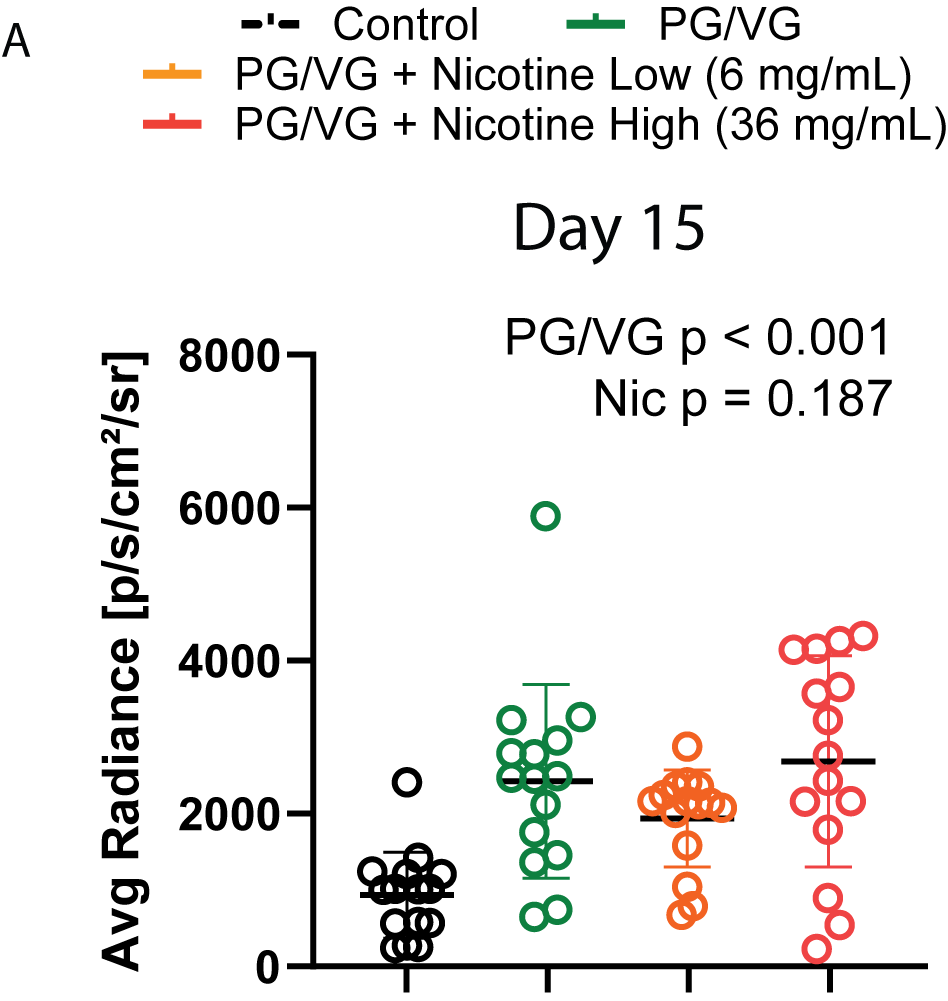

Supplement: Supplementary Figure 3 — PG/VG tumor cell preconditioning induces more aggressive Luc-MC38 tumors. (A) Tumor bioluminescence on day 15 after implantation expressed as average photon radiance. Tumor bioluminescence was significantly increased in cells preconditioned with PG/VG (p<.001; Table 1 ). Adding nicotine had no additional effect (p=0.187). [file Image3.tif]

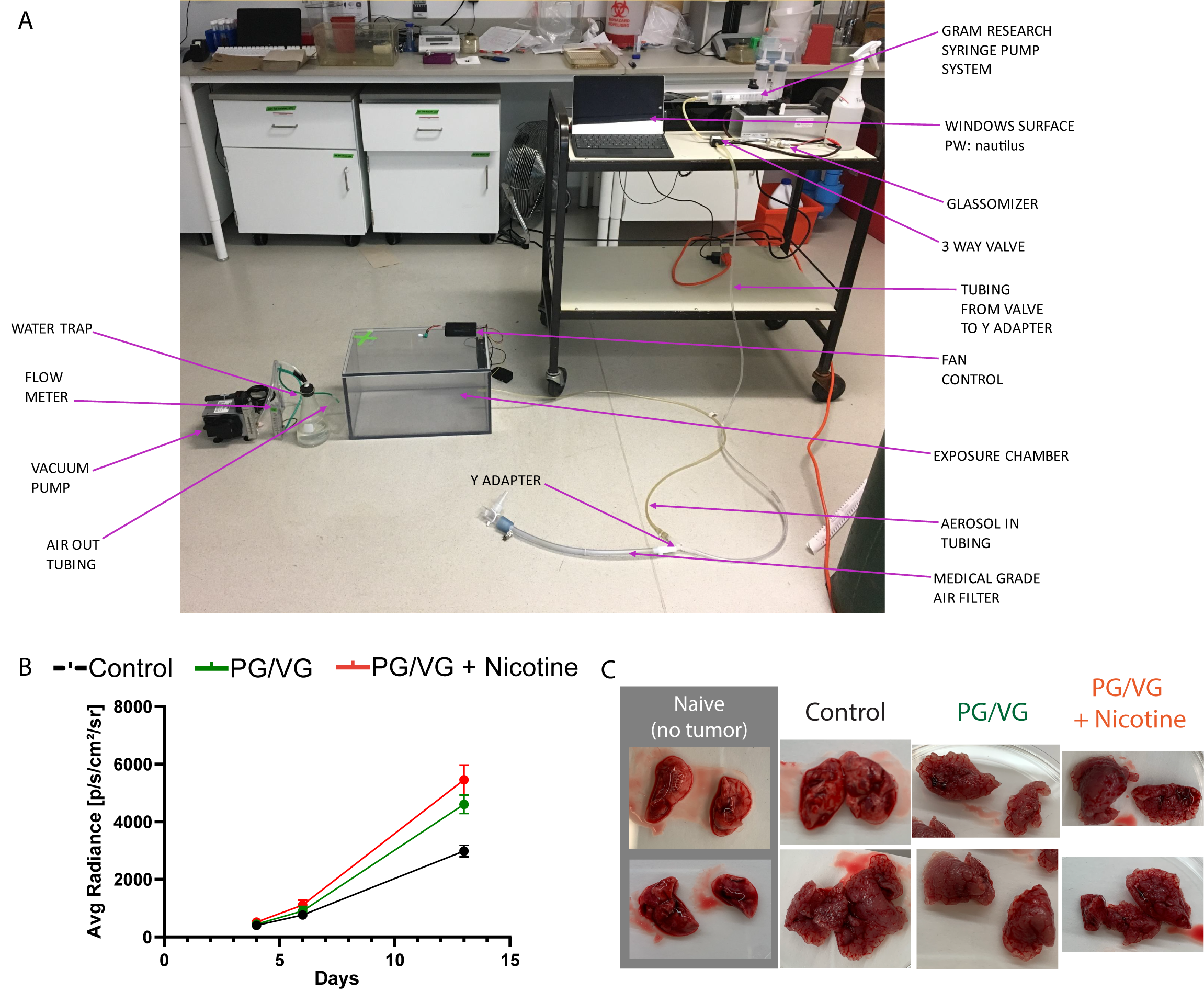

Supplement: Supplementary Figure 4 — Whole body exposure of mice e-cigarette aerosol leads accelerated tumor growth and more aggressive metastasis. (A) Whole body exposure machine set up for experiments described in Figure 2 . Briefly, vaped e-cigarette liquids were injected into an exposure chamber containing live mice continuously for 1h, 5 days a week. (B) Time-course measurement of tumor bioluminescence expressed as Average Radiance for mice involved in experiments described in Figure 2 . Error bars represent SEM. (C) Representative pictures of harvested lungs from unchallenged mice (left) or e-cigarette-exposed mice (right). [file Image4.tif]

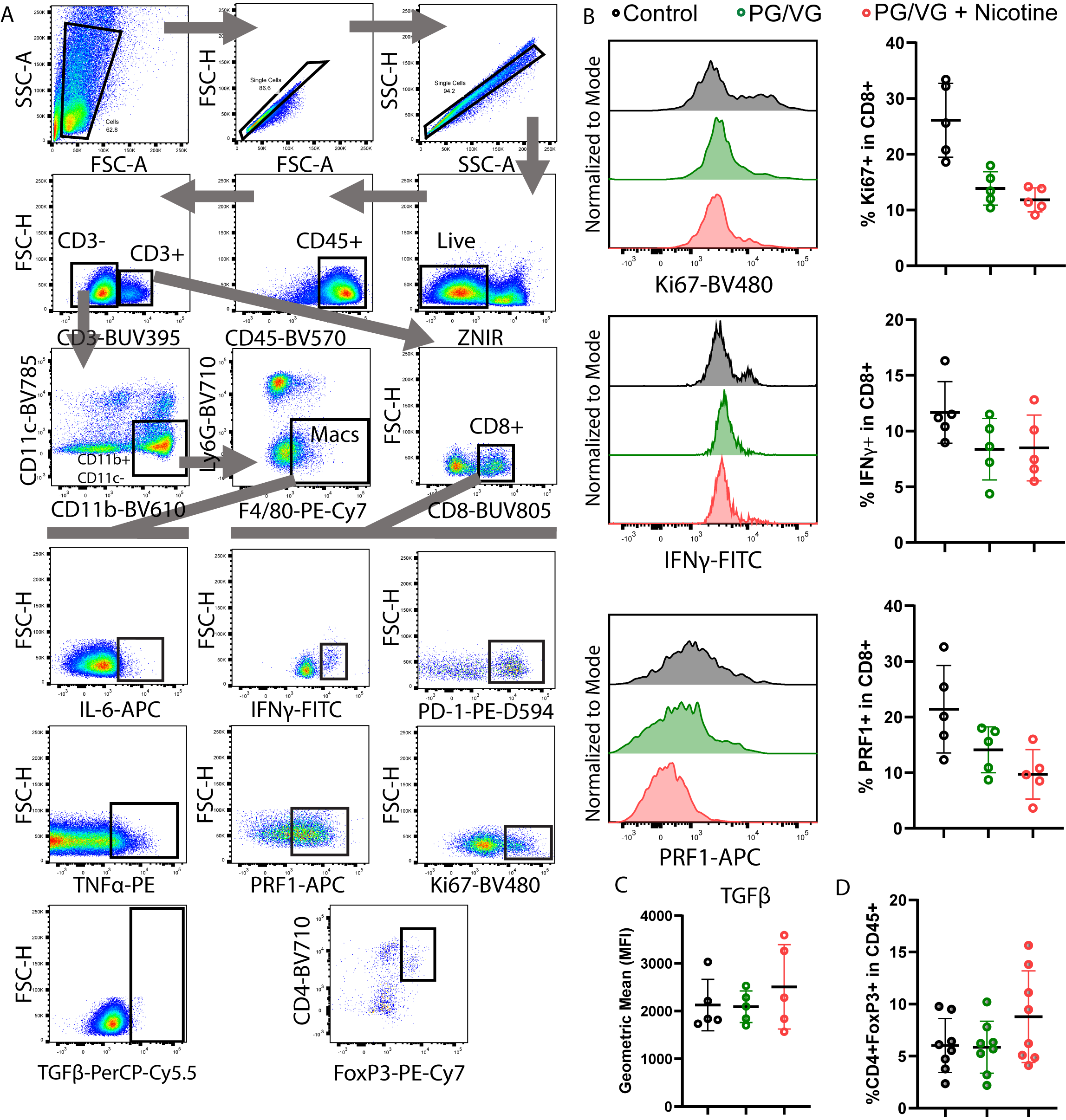

Supplement: Supplementary Figure 5 — Whole body exposure of mice e-cigarette aerosol leads to increased myeloid and lymphoid immunosuppression in the tumor microenvironment. (A) Flow cytometry gating strategy used for phenotyping of Luc-MC38 tumor immune infiltrates harvested from lungs of mice involved in experiments described in Figure 2 . (B) Left, offset histograms representing normalized modal frequencies for markers Ki67, IFNγ and PRF1 in gated tumor-infiltrating CD8+ T cells. Right, percentages of Ki67+, IFNγ+ and PRF1+ T cells within the CD8+ compartment. (C) Mean fluorescence intensity of TGFβ in gated lung macrophages was not affected significantly (p=0.5257). (D) Percentage CD4+FoxP3+ cells in gated CD45+ cells. No significant differences were observed (p=0.1597). [file Image5.tif]

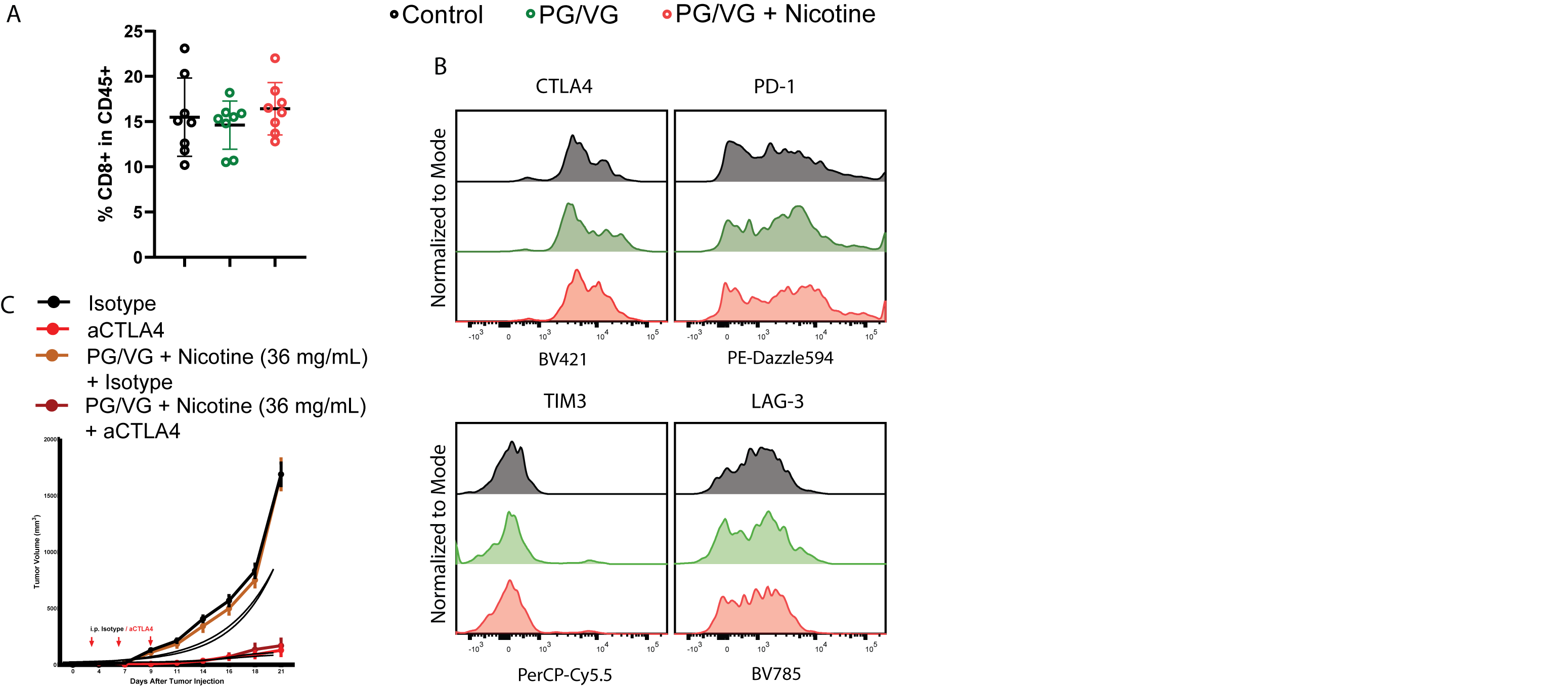

Supplement: Supplementary Figure 6 — Tumors exposed to proinflammatory e-cigarette components are responsive to immune checkpoint blockade. (A) Splenic CD8+ T cell frequencies within gated live CD45+ immune cells in mice involved in whole body exposure experiments. (B) Representative flow cytometry histograms showing normalized modal frequencies for CTLA4, PD-1, TIM3 and LAG-3 expression in gated splenic CD8+ T cells. (C) Tumor volume curves for n=3 experiments described in Figure 4 . Red arrows indicate Isotype control or anti-CTLA4 treatment. Black lines represent fit curves. Error bars represent SEM. [file Image6.tif]
