# Supplementary material for: E-cigarette exposure disrupts antitumor immunity and promotes metastasis
Source: Front Immunol. 2024 Aug 16;15:1444020. doi: 10.3389/fimmu.2024.1444020 (PMC11365074; doi:10.3389/fimmu.2024.1444020)
Supplement: Supplementary Table 1 — Antibodies used for flow cytometry. [file Table1.docx]

| **Marker** | **Conjugation** | **Vendor** | **Catalog #** | **Clone** |
| --- | --- | --- | --- | --- |
| CD45 | BV570 | Biolegend | 103136 | 30-F11 |
| CD8α | BUV805 | BD | 612898 | 53-6.7 |
| CD3 | BUV395 | BD | 563565 | 145-2C11 |
| CD4 | BV711 | Biolegend | 100447 | GK1.5 |
| CD11b | BV605 | Biolegend | 101237 | RTK4530 |
| CD11c | BV785 | Biolegend | 117335 | N418 |
| Ly6G | BV711 | Biolegend | 127643 | 1A8 |
| F4/80 | PE-Cy7 | Biolegend | 123114 | BM8 |
| FoxP3 | PE-Cy7 | Life Tech | 25-5773-80 | FJK-16s |
| PD-1 | PE-Dazzle594 | Biolegend | 109115 | RMP1-30 |
| TNFα | PE | Biolegend | 506305 | MP6-XT22 |
| IL-6 | APC | Biolegend | 504507 | MP5-20F3 |
| PRF1 | APC | Biolegend | 154303 | S16009A |
| IFNγ | FITC | BD | 554411 | XMG1.2 |
| Ki67 | BV480 | BD | 566109 | B56 |
| CTLA4 | BV421 | Biolegend | 106312 | UC10-4B9 |
| TIM3 | PerCP-Cy5.5 | Biolegend | 134012 | B8.2C12 |
| TGFβ | PerCP-Cy5.5 | Biolegend | 141409 | TW7-16B4 |
| LAG-3 | BV785 | Biolegend | 125219 | C9B7W |

**Supplementary Table 1. Antibodies used for flow cytometry.**
